# Genetic Determinants of Chronic Kidney Disease (CKD) in India: A Comprehensive Genomewide Association Study (GWAS) Analysis

**DOI:** 10.1155/ijne/5578625

**Published:** 2025-11-20

**Authors:** Mythri Shankar, Sairam Bashyam, Gireesh Reddy, Kishan A., Sreedhara C. G.

**Affiliations:** Department of Nephrology, Institute of Nephrourology, Bengaluru, India

**Keywords:** chronic kidney disease, genomewide association studies, Indian population

## Abstract

**Introduction:**

Chronic kidney disease (CKD) poses a major health burden globally and affects nearly 17% of the Indian population. Despite established risk factors such as diabetes and hypertension, significant interindividual variability suggests a genetic contribution to CKD susceptibility. This study explores genetic variants predisposing to CKD in the Indian population using a genomewide association approach.

**Methods:**

A total of 90 patients with CKD and 90 healthy controls were genotyped using the Illumina Infinium Global Screening Array (640,000 markers). After stringent quality control, 5.7 million genetic markers were retained for analysis. Single-nucleotide polymorphisms (SNPs) were assessed using logistic regression including age, sex, and ten principal components as covariates. Variants meeting standard genomewide significance thresholds (*p* ≤ 5 × 10^−8^) were considered significant.

**Results:**

The study identified 87 SNP loci associated with CKD, of which two genes, MPP7 and MAD1L1, reached genomewide significance. Variants with extremely high odds ratios (e.g., MAD1L1 OR > 1000) were interpreted as possible methodological artifacts. SNPs in PDZK1IP1, ANO3, C3AR1, FTO, and CD70 demonstrated suggestive biomarker potential (OR < 10, *p* < 10^−4^), warranting replication in larger cohorts. These findings provide new insights into genes involved in tubular integrity, immune activation, and metabolic regulation in CKD.

**Conclusion:**

This genomewide analysis represents one of the first studies on CKD genetics in the Indian population. While the MPP7 variant emerges as a credible susceptibility locus, several other SNPs show promising biomarker potential. Larger, multiethnic studies and functional validation are needed to confirm their roles in CKD pathogenesis and therapeutic targeting.

## 1. Introduction

Chronic kidney disease (CKD) is a major public health problem with a global prevalence of 9.5%, and in India, it is 17.2% [1–3]. Incidence and prevalence have been increasing worldwide. Eventually, these CKD patients progress to end-stage kidney disease (ESKD) requiring kidney replacement therapy. There are over 175,000 patients receiving dialysis, and the number is increasing by 232 per million population [4]. The high prevalence of CKD with its increased morbidity and mortality is a major public health problem.

Though the prevalence of CKD has been increasing, our understanding of the risk factors and pathogenesis for CKD is limited [5]. Diabetes and hypertension are important and well-known risk factors [6]. Even in the setting of diabetes and hypertension, there is marked variability in the development of CKD and its progression to ESKD [7]. Also, studies have shown important genetic contributions toward increasing the risk of developing CKD and its progression to ESKD [8, 9]. Kottgen et al. used genomewide association genes and identified 3 gene loci in UMOD, SHROOM3, and STC1 associated with CKD among participants of European ancestry [10]. Another study among Caucasian individuals found that 13 more loci along with the three already identified were associated with CKD [11]. One other study among the Spanish population analyzed 38 single-nucleotide polymorphisms (SNPs) among candidate genes associated with fibrosis, inflammation, renin–angiotensin–aldosterone system activation, antioxidant system, homocysteine synthesis, and Phase II metabolism [12]. A page study from the USA identified that NMT2 and APOL1 loci were associated with CKD [13]. There are no studies so far on the Indian population. By identifying the susceptibility loci, we can screen the population to identify individuals at risk for CKD and reduce the morbidity and mortality associated with it through early diagnosis and targeted therapy. Also, by identifying the genetic loci, we can understand the underlying pathogenetic mechanism causing CKD and target therapy accordingly.

### 1.1. Aim of the study

To determine the possible association between allelic variants and susceptibility to CKD in the Indian population.

## 2. Methodology

### 2.1. Ethics Statement

All the participants gave informed consent, and blood samples were collected under the protocol approved by the institutional ethics committee in accordance with the Declaration of Helsinki. The blood sample was used for genotyping and standard biochemical tests relevant to CKD.

### 2.2. Study Population

A pilot study of 90 cases and 90 controls was conducted in a tertiary care hospital in South India between January 2022 and December 2023.

### 2.3. Cases

South Indian population at varying stages of CKD, i.e, structural or functional abnormality of the kidney (eGFR < 60 mL/min/1.73 m^2^) persisting for 3 months or more.

### 2.4. Control

Matching controls from the South Indian population with normal kidney function tests (normal eGFR according to age, normal kidneys on USG, and normal urine routine and microscopy examination) from the Medgenome database [14].

Sample size was calculated taking into account the prevalence of CKD in the state, which is 6.2% [15] of the entire population, 95% confidence level, and 5% margin of error.

Exclusion criteria: Patients with a concomitant diagnosis of malignancy, chronic hepatic disease, and autoimmune disease.

## 3. Methodology

### 3.1. DNA Extraction and Genotyping

Genomic DNA was extracted from whole blood by magnetic separation using the QIA Symphony automated DNA isolation system (QIAGEN, Hilden, Germany) following the manufacturer's protocol. Genomic DNA quantification was performed on the Thermo Scientific Multiskan Sky Microplate Spectrophotometer (ThermoFisher Scientific, USA). The quality of the DNA was checked on an agarose gel. Whole genome genotyping was performed using Infinium Global Screening Array-24 V3.0 BeadChip (Illumina, Inc, San Diego, California), containing probes that cover ∼640 k markers. Samples were processed according to the recommendations of the Illumina Infinium HTS assay. In brief, two hundred nanograms of genomic DNA were isothermally amplified at 37°C for 20–24 h in an Illumina hybridization oven. The bead chips were placed into the hybridization chambers and incubated in an Illumina hybridization oven at 48°C for 16–24 h. After hybridization, the chips were washed and prepared for single-base extension. After single-base extension, the chips were stained with fluorescent dyes (biotin for G and C nucleotides and DNP for A and T nucleotides) and scanned using the iScan system. Normalized bead intensity data gained for each sample were run through the GenomeStudio software (Illumina, Inc, San Diego, California) to obtain SNP genotype calls from the fluorescence intensities.

### 3.2. Genomewide Association Study (GWAS) Analysis

The GWAS analysis was performed on a total of 180 samples, including 90 cases and 90 MedGenome database controls.

Data QC was performed, and a genotyping cutoff of 0.95 was applied to filter out low-quality samples. Quality control measures were applied to the dataset, including minor allele frequency (MAF) filtering at 1% and Hardy–Weinberg equilibrium (HWE) testing at a *p* value threshold of 1e − 4. A dosage allelic r2 filter of 0.2 was applied in the imputed data.

After quality control, a total of 5,774,845 markers remained for further analysis (Supporting file 2).

Genotype imputation was performed using the Beagle 5.0 tool with a reference panel consisting of phased high-coverage whole genome data of 6461 South Asian samples, covering 70+ South Asian ethnicities/regions. The imputed VCF has ∼25 million sites.

The association (GWASs) was performed using age, gender, and the top 10 principal components as covariates. The significant markers obtained were annotated.

Major tools used were PLINK (Version 2.0), VCFtools (Version 0.1.14), Tabix (Version 0.2.6), and Python (Version 2.7).

Principal component analysis (PCA) was performed to correct for population stratification, which refers to differences in allele frequencies due to ancestry. PCs are derived from genotype data and capture major sources of genetic variation in the dataset. Typically, the first few PCs are used as covariates in association studies (e.g., GWAS) to reduce confounding due to ancestry differences.

## 4. Results

The mean age of cases was 54.9 ± 10.6 years, with the majority being males (71.11%). The baseline characteristics of cases and controls are described in Tables 1 and 2. There was no significant difference noted between cases and controls with respect to the parameters elaborated in Table 1.

**Figure 1:**
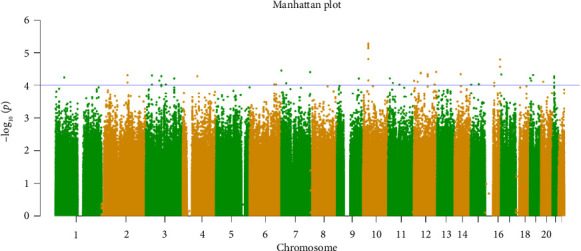
This Manhattan plot suggests that there are SNPs associated with chronic kidney disease. Several genomic regions (on various chromosomes) show statistically significant associations as they rise above the threshold line (blue horizontal line). The highest peaks may point to candidate genes or regions for further investigation.

**Figure 2:**
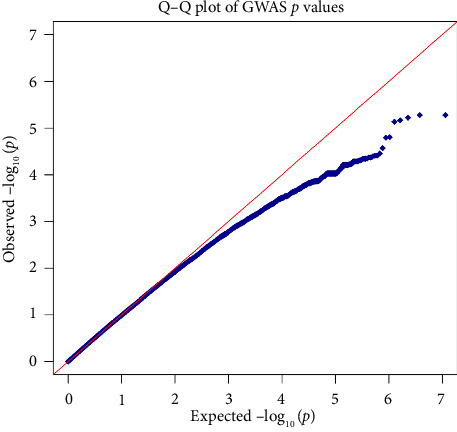
Quantile–quantile (Q-Q) plot is to compare observed (blue diagonal line) versus expected (red diagonal line) distributions of *p* values for each SNP. This Q–Q plot indicates that the SNPs that lie along the red line do not show significant associations. However, the upward deviations at higher −log_10_ (*p*) values suggest that there are some SNPs with significant associations to chronic kidney disease. The plot confirms that the GWAS has identified a number of SNPs with potential true associations with CKD.

A GWAS was conducted to identify significant genetic markers associated with the CKD. The Manhattan plot (Figure 1) and the Q–Q plot (Figure 2) confirm that there were genes that were significantly associated with CKD in the Indian population.

87 loci across multiple chromosomes were identified to be significantly associated with CKD with a *p* value of < 1 × 10^4^ (Supporting Table 1). With a standard stringent GWAS threshold [16] of *p* value < 5 × 10^8^, we found two significant genes:

• Membrane protein, palmitoylated 7 (MPP7)
• MAD1L1

The observation of an extremely high odds ratio (> 1000) for MAD1L1 likely reflects a methodological limitation of the study, rather than a true biological effect. Such inflated effect sizes can result from factors such as small sample size, rare variant frequency, or statistical artifacts, which are known to exaggerate associations in genomewide studies involving limited cohorts. Hence, this variant was not considered for further evaluation.

Functional impact of SNPs (Table 3, Supporting Table 1): The SNPs were located in different genomic regions, affecting disease risk in various ways:

○ 5′ UTR or regulatory regions: Variants in regions such as the 5′ UTR (such as rs7519456 in PDZK1IP1) can affect how genes are expressed, possibly altering protein production and contributing to disease development.
○ Intronic SNPs: SNPs located in noncoding regions (such as introns) can still affect gene regulation by influencing splicing, transcription factor binding, or gene expression (e.g., rs17029455 in CMTM7).
○ Intergenic SNPs: Variants in intergenic regions may affect regulatory elements (like enhancers), even though they are far from the gene itself.

Population-specific allele frequencies (Table 2): The MAFs observed in different populations, such as those provided by the gnomAD dataset, indicate how common or rare a SNP is in various ancestry groups. For example,

○ SNP rs7519456 has a higher frequency in European populations (gnomAD_NFE_AF = 0.6943) than in South Asian populations (gnomAD_SAS_AF = 0.5002). This could imply that its contribution to disease risk might differ across populations due to the variant's prevalence.

Understanding population-specific allele frequencies is crucial for assessing disease risk in different demographic groups and tailoring prevention or treatment strategies.

Gene involvement and pathways (Table 3): The involvement of specific genes near or affected by the significant SNPs can provide insights into the biological mechanisms driving disease risk.

Biomarker potential: For this study, considering international GWAS best practices and the nature of the dataset, it would be reasonable to set the odds ratio threshold for excluding SNPs/genes at around 10. Odds ratios above this value are rare for common variants in complex diseases and are much more likely to reflect methodological artifacts or statistical anomalies rather than true biological associations.

Among the SNPs with an odds ratio less than 10 in this study, the following SNPs have biomarker potential based on their statistical significance and annotation:

• rs7519456 (PDZK1IP1): Odds ratio 5.29, *p* value 5.71 × 10^−5^.
• rs721258 (anoctamin-3 [ANO3]): Odds ratio 3.87, *p* value 8.50 × 10^−5^.
• rs2230318 (C3AR1): Odds ratio 8.87, *p* value 7.28 × 10^−5^.
• rs12599672 (FTO): Odds ratio 0.13, *p* value 8.32 × 10^−5^.
• rs12610000 (CD70): Odds ratio 4.98, *p* value 7.17 × 10^−5^.

However, none of these SNPs reach the stringent GWAS significance threshold of 5 × 10^−8^. Thus, while their associations are statistically noteworthy and may inform biomarker screening in early research or pilot clinical assays, validation in larger cohorts is necessary before they are accepted as robust disease biomarkers.Based on our dataset, SNPs with odds ratios under 10 and notable statistical associations include the following:

• rs7519456 (PDZK1IP1)
• rs721258 (ANO3)
• rs2230318 (C3AR1)
• rs12599672 (FTO)
• rs12610000 (CD70)

These SNPs have *p* values below 0.0001 and odds ratios within the plausible biomarker range (< 10), but none meet the genomewide significance threshold of 5 × 10^−8^, so they are best considered candidates for exploratory biomarker research until they are independently validated in larger studies [17].

## 5. Discussion

In recent times, advancements in genetic analysis techniques have helped clarify previously uncertain areas, emphasizing the role of genetic factors as a significant, yet often overlooked, contributor to CKD. There are two primary types of genetic kidney disorders: monogenic (or Mendelian) and polygenic. Monogenic nephropathies, resulting from mutations in single genes, have emerged as a significant but often underrecognized cause of CKD. Current research suggests that these genetic disorders account for approximately 70% of ESKD cases in children and between 10% and 15% of adult ESKD cases [15, 16]. Despite the significance of monogenic diseases, their contribution to the overall public health burden of CKD is relatively modest. This suggests that additional genetic elements, likely involving more complex interactions, play a role in the rising incidence of CKD within the general population. These findings highlight the need to explore beyond single-gene mutations to understand the broader genetic landscape influencing CKD prevalence.

Polygenic nephropathy refers to a genetic susceptibility to kidney disease that often manifests in conjunction with other conditions, such as diabetes mellitus, which carries a high risk for kidney damage. This predisposition is influenced by multiple genetic factors. To date, numerous genetic loci and SNPs have been linked to kidney function traits such as glomerular filtration rate (GFR) and albuminuria, as well as to distinct forms of CKD [11–13]. These findings highlight the complex genetic architecture that contributes to CKD risk.

The introduction of advanced genetic analysis methods, such as GWASs, has significantly advanced the field of nephrogenetics. These techniques have uncovered new genetic loci and shed light on previously unidentified mechanisms responsible for kidney damage. Unlike traditional approaches that target specific genes, GWAS takes an unbiased, genomewide approach, scanning for associations between genetic variants, such as SNPs, and CKD. By comparing the frequency of SNPs in CKD patients versus control groups, we can pinpoint genetic loci potentially linked to CKD.

GWAS has led to the discovery of genetic loci potentially involved in the mechanisms of kidney damage, offering new insights that could guide future therapeutic strategies [17].

Most of the genetic variants identified through GWAS that are linked to kidney function are predominantly expressed in the proximal tubules of the kidneys. This supports a tubule-centered perspective of both acute and chronic kidney injury, highlighting the proximal tubules as the primary site of damage [18–20]. As a result, the progression of kidney disease appears to largely stem from injuries targeting this particular part of the kidney. This is in concordance with this study as well. The significant genes are discussed as follows and shown in Tables 3 and 4.

The gene MPP7 is a member of the membrane-associated guanylate kinase (MAGUK) family of proteins, which are involved in maintaining cell polarity and tight junction formation in epithelial cells. Tight junctions play a crucial role in regulating the permeability of the epithelial barrier, including in the kidneys. Loss of cell polarity and tight junction integrity is a hallmark of many kidney diseases, including focal segmental glomerulosclerosis (FSGS), diabetic nephropathy, and acute kidney injury (AKI) [23].

Gene PDZK1IP1 (MAP17): MAP17 and Gene PDZK1IP1 refer to the same protein, meaning there is no functional difference between them; MAP17 is simply the protein's common name, while PDZK1IP1 is the gene that encodes for that protein, essentially the genetic identifier for MAP17; both terms indicate a small, membrane-associated protein that interacts with PDZK1 and is often overexpressed in various cancers. The renal proximal tubule plays a key role in glucose reabsorption, handling 90% of the filtered glucose through the sodium–glucose transporter SGLT2. Research using expression cloning identified MAP17 (gene PDZK1IP1), a 17 kDa membrane-associated protein, that significantly enhances SGLT2 activity by up to 100-fold when coexpressed in certain cells, such as Xenopus oocytes and opossum kidney cells. However, this increased activity was not due to higher amounts of SGLT2 on the cell surface, indicating that MAP17 enhances the transporter's function without affecting its expression levels.

Further studies in a cohort of patients with familial renal glucosuria, a condition where glucose is excreted in the urine, revealed that one patient had a splicing mutation in the MAP17 gene (PDZK1IP1) rather than a mutation in the SGLT2 gene itself. This underscores the physiological relevance of the MAP17–SGLT2 interaction. These findings pave the way for further exploration of SGLT2's regulation and potential effects on other renal transporters when inhibited [24].

**CD70**, a member of the tumor necrosis factor (TNF) family, is primarily expressed on activated T cells and other immune cells. CD70 binds to its receptor, CD27, facilitating T-cell activation, differentiation, and survival, and plays a key role in inflammatory responses. CD70 has been implicated in promoting inflammatory responses in autoimmune kidney diseases such as lupus nephritis and glomerulonephritis. Due to its involvement in immune activation, CD70 is being explored as a therapeutic target [25].

The fat mass- and obesity-associated (FTO) gene has primarily been studied for its role in regulating body mass and obesity, but recent research has begun to explore its broader impacts on metabolic diseases, including potential links to kidney disease. Obesity increases the risk of hypertension, diabetes, and glomerular hyperfiltration, all of which can contribute to CKD [26].

ANO3: The identification of ANO3 as a CKD-associated locus in this study is particularly noteworthy given its key biological role in the kidney. ANO3 encodes a calcium-activated chloride channel expressed in renal epithelia, where it facilitates glomerular filtration and tubular reabsorption, thereby maintaining electrolyte homeostasis. Variations in ANO3 could disrupt this finely tuned electrolyte balance, so the intronic ANO3 variant uncovered in our GWAS may contribute to CKD susceptibility and serve as a genetic marker for early detection of at-risk individuals. Notably, previous CKD genetic studies have seldom implicated ANO3, making our finding a novel insight, though related chloride channel genes such as ANO9 have been linked to kidney disease risk, highlighting the broader importance of electrolyte transport pathways. Understanding ANO3's involvement in CKD also opens therapeutic avenues: if ANO3-mediated chloride flux is indeed influencing disease progression, it could be targeted by drugs aimed at restoring electrolyte homeostasis, and patients carrying ANO3 risk alleles might benefit from precision medicine strategies tailored to their genetic profile [27]. Our identification of novel genetic loci, such as ANO3, contributing to CKD susceptibility aligns with findings from large-scale genomic studies such as those by Stanzick et al. [28].

C3AR1: GWASs have implicated the C3AR1 locus in kidney disease risk and renal function. Large meta-analyses of kidney function (e.g., CKDGen, > 1 million individuals) have identified hundreds of loci associated with eGFR and CKD, including a region on Chromosome 12p13 that harbors *C3AR1* [29, 30]. In one GWAS of the European population, a lead SNP in this region (e.g., rs10774021 on 12p13) showed genomewide significance for CKD (*p*∼1 × 10^9^) [31]. Notably, this 12p13 locus contains multiple genes; initial studies highlighted nearby genes (*JARID1A/KDM5A* and *SLC6A12/13*), but C3AR1 is also located in this interval and is a plausible contributor given its role in complement activation. Recent GWAS meta-analyses have reinforced the association of this locus with renal traits, suggesting that C3AR1 or linked variants influence CKD susceptibility [29].

### 5.1. Strengths of the Study

The identification of risk variants provides insight into the biological pathways of CKD, potentially pointing to new therapeutic targets. As noted by Fountoglou et al., GWASs have been instrumental in reconstructing the complex genetic basis of CKD, offering insights into potential biomarkers and therapeutic targets [32]. This study contributes to this expanding field by exploring underrepresented populations, thereby enriching the genetic datasets crucial for global comparisons and therapeutic advancements.

### 5.2. Limitations of the Study

The data predominantly come from a South Asian population; hence, the findings may not be generalizable to populations of other ancestry. The study does not account for environmental or lifestyle factors, which are critical in modulating disease risk. Larger sample sizes are needed to identify variants with small effect sizes and to reduce the likelihood of false positives.

## 6. Conclusion

This study provides new genetic insights into CKD within an Indian population, identifying 87 loci associated with CKD risk. Notably, one gene, **MPP7**, achieved genomewide significance, underscoring its potential role as a key susceptibility locus. While several additional SNPs displayed biomarker promise, further large-cohort validation is necessary to confirm their utility. The results highlight the polygenic and complex nature of CKD and reinforce the need for expanded, multicenter research to enable advances in early diagnosis and precision medicine targeting these newly identified genetic determinants.

## Figures and Tables

**Table 1:** Comparison of cases and controls.

Baseline parameter	Cases *n* = 90 (100%)	Controls *n* = 90 (100%)
Mean age ± SD (years)	54.9 ± 10.6	58 ± 6.4
Males	64 (71.11%)	70 (77.77%)
Females	26 (28.89%)	20 (22.22%)
Diabetes mellitus	58 (64.44%)	50 (55.55%)
Hypertension	90 (100%)	90 (100%)
Other comorbidities		
Coronary artery disease	20 (22.22%)	17 (18.89%)
Hypothyroidism	16 (17.77%)	12 (13.33%)
Cerebrovascular accident	1 (0.11%)	0 (0%)
Parkinsonism	1 (0.11%)	0 (0%)
Obesity	1 (0.11%)	0 (0%)
Duration of DM (mean ± SD years)	5.61 ± 2.82	5.1 ± 4.32
Duration of HTN (mean ± SD years)	4.12 ± 2.88	3.8 ± 3.78

**Table 2:** Baseline characteristics of cases with chronic kidney disease.

Medication for HTN	No. of patients (*n* = 90)	%
Amlodipine	66	73.3
Metoprolol	32	35.6
Nifedipine	14	15.6
Telmisartan	10	11.1
Arkamin	4	4.4
Prazosin	4	4.4
Ramipril	3	3.3

**Oral hypoglycemic drugs**	**No. of patients**	**%**

Linagliptin	23	25.6
Teneligliptin	17	18.9
Insulin	21	23.3

**Lab parameters**	**Mean**	**Standard deviation**

HBA1C (g%)	6.663	0.845
Hemoglobin (g%)	9.944	1.005
Total count (cells/mm^3^)	8166.956	2075.080
Platelet count (cells/mm^3^)	2.570	0.696
S. Creatinine (mg/dL)	4.266	2.980
Total protein (g/L)	6.596	0.503
S. Albumin (g/L)	3.361	0.442

**Urine protein dipstick**	***n***	**%**

1+	17	18.9
2+	46	51.1
3+	19	21.1
Trace	8	8.9

**24-h urine protein (g)**	***n***	**%**

< 1.0	12	13.3
1.0–5.0	68	75.6
> 5.0	10	11.1
Diabetic retinopathy	23	25.6
Hypertensive retinopathy	37	41.1
Neuropathy	37	41.1

**Table 3:** Functional impact of SNPs: the SNPs can be located in different genomic regions, affecting disease risk in various ways.

Chrom	Start	Ref	Alt	A1	Odds ratio	*p* value	Gene name	dbSNP_rsid	Varclass
chr1	47656575	G	A	A	5.29	0.00006	PDZK1IP1	rs7519456	5UTR
chr10	28389777	A	C	C	14.93	0.00001	MPP7	rs11006868	Intronic
chr10	28390053	T	C	C	19.43	0.00001	MPP7	rs79324627	Intronic
chr10	28391979	C	G	G	14.93	0.00001	MPP7	rs75177557	Intronic
chr10	28395057	T	A	A	19.58	0.00001	MPP7	rs58590537	Intronic
chr10	28399956	T	C	C	14.95	0.00001	MPP7	rs76071434	Intronic
chr10	28405736	T	A	A	16.67	0.00002	MPP7	rs78302884	Intronic
chr10	28409394	G	A	A	16.16	0.00007	MPP7	rs76927140	Intronic
chr11	26508587	A	T	T	3.86	0.00008	ANO3	rs721258	Intronic
chr12	8211256	C	T	T	8.86	0.00007	C3AR1	rs2230318	3UTR
chr16	54116076	T	A	A	0.13	0.00008	FTO	rs12599672	Intronic
chr19	6582133	C	T	T	4.97	0.00007	CD70	rs12610000	3UTR
chr20	12886331	C	T	T	0.12	0.00008	RP5-1069C8.2	rs6041707	Intronic

*Note:* Chrom, variant chromosome number; Start, variant start position in the chromosome (hg19); Ref, reference nucleotide at the variant position (hg19); Alt, observed nucleotide (also referred as alt base) in the sample; A1, the minor allele; Odds ratio, measure of how strongly an event is associated with the exposure; *p* value, the probability under the assumption of no effect or no difference in obtaining a result equal to or more extreme than what was actually observed; Gene name, HUGO gene symbol from Ensembl; dbSNP_rsid, dbSNP variant identifier; Varclass, variant type classification.

**Table 4:** Significant genes involved in CKD and their functions.

Gene	Alternative titles/symbols	Description	Disease associations
PDZK1-interacting protein 1; PDZK1IP1	Membrane-associated protein 17; MAP17	It is a gene that encodes a protein involved in various cellular processes, including protein–protein interactions and signal transduction pathways	Kidney carcinoma
	DD96		

CD70	CD27 ligand; CD27L	CD70 binds to its receptor, CD27, facilitating T-cell activation, differentiation, and survival, and plays a key role in inflammatory responses	Lupus nephritits, Glomerulonephritis, kidney transplant rejection.
	Tumor necrosis factor ligand superfamily, member 7; TNFSF7		

FTO alpha-ketoglutarate-dependent dioxygenase; FTO	FAT mass- and obesity-associated GENE	In genomewide association studies of Type 2 diabetes involving genotype data from a variety of international consortia, Zeggini et al. [21] and Scott et al. [22] reported FTO as a diabetes susceptibility locus	Obesity, severe growth retardation, developmental delay, and facial dysmorphism
	FATSO, mouse, homolog of AlkB homolog 9; ALKBH9		

MPP7 membrane protein, palmitoylated 7; MPP7		Membrane-associated guanylate kinases (MAGUKs) are important adapter proteins involved in the assembly of protein complexes at sites of cell–cell contact. They are found in synapses, adherens junctions, and tight junctions	Diabetic nephropathy, focal segmental glomerulosclerosis

Anoctamin 3; ANO3	Transmembrane protein 16C; TMEM16C	The ANO3 gene encodes anoctamin-3, a transmembrane protein that belongs to a family of calcium-activated chloride channels involved in glomerular filtration and tubular reabsorption	Electrolyte imbalance, chronic kidney disease
	Chromosome 11 open reading frame 25; C11ORF25		

Complement component 3a receptor 1; C3AR1	C3AR	The C3AR1 gene encodes the C3a receptor 1, a G-protein–coupled receptor that interacts with C3a, a component of the complement system. The complement system is a key part of the immune system that helps clear pathogens and damaged cells, but its dysregulation can lead to inflammation and tissue damage, including in the kidneys	Glomerulonephritis, lupus nephritis, and atypical hemolytic uremic syndrome (aHUS), diabetic nephropathy'

## Data Availability

The data that support the findings of this study are openly available in Zenodo at https://zenodo.org/records/15032222?preview=1%26token=eyJhbGciOiJIUzUxMiJ9.eyJpZCI6ImQyYzM0M2Q4LTVhMzgtNDA2MC05ZDE3LWQyNjhkNDExMmFlYyIsImRhdGEiOnt9LCJyYW5kb20iOiJmNzQ1N2NkNjI0MTFlZGY0MDk1ZjJlZWQ4YzFkZTUzOCJ9.RLFL1Z-SdfVhs_xkDMJO-5w32mviF2LM8x3G8kZdizIDtX2CS. Related files: Raw data.

## Supporting Information

Supporting Information 1 Supporting Table 1 presents the list of significant genetic markers identified in the association analysis, annotated with detailed variant information. Each entry includes chromosome location, variant start position (hg19), reference and alternative alleles, minor allele, odds ratio, *p* value, gene symbol, dbSNP variant identifier, variant classification, and comprehensive allele frequencies from gnomAD across diverse populations. Where applicable, GWAS-reported disease associations and TOPMed allele frequencies are also included for reference. This table provides a resource for the interpretation of variant-level findings from the study and their population-level context.

Supporting Information 2 Supporting file 2: Quality check report.
