# Supplementary material for: Genetic Determinants of Chronic Kidney Disease (CKD) in India: A Comprehensive Genomewide Association Study (GWAS) Analysis
Source: Int J Nephrol. 2025 Nov 20;2025:5578625. doi: 10.1155/ijne/5578625 (PMC12660636; doi:10.1155/ijne/5578625)
Supplement: Supporting Information 2 — Supporting file 2: Quality check report. [file 5578625.f2.pdf]

Genotype Array QC Report

Project Name- Ancestry

Project ID- Batch1

Analyst Name- Lekha

MedGenome Labs Ltd.

July 30, 2024

Contents

1 Array QC **2**
1.1 Sample QC report . . . 2
1.1.1 Missing genotype and heterozygosity . . . 2
1.1.2 Genotyping rate distribution . . . 3
1.1.3 Principal Component Analysis . . . 4
1.1.4 Genotype Rate Distribution table . . . 5

1 Array QC

1.1 Sample QC report

1.1.1 Missing genotype and heterozygosity

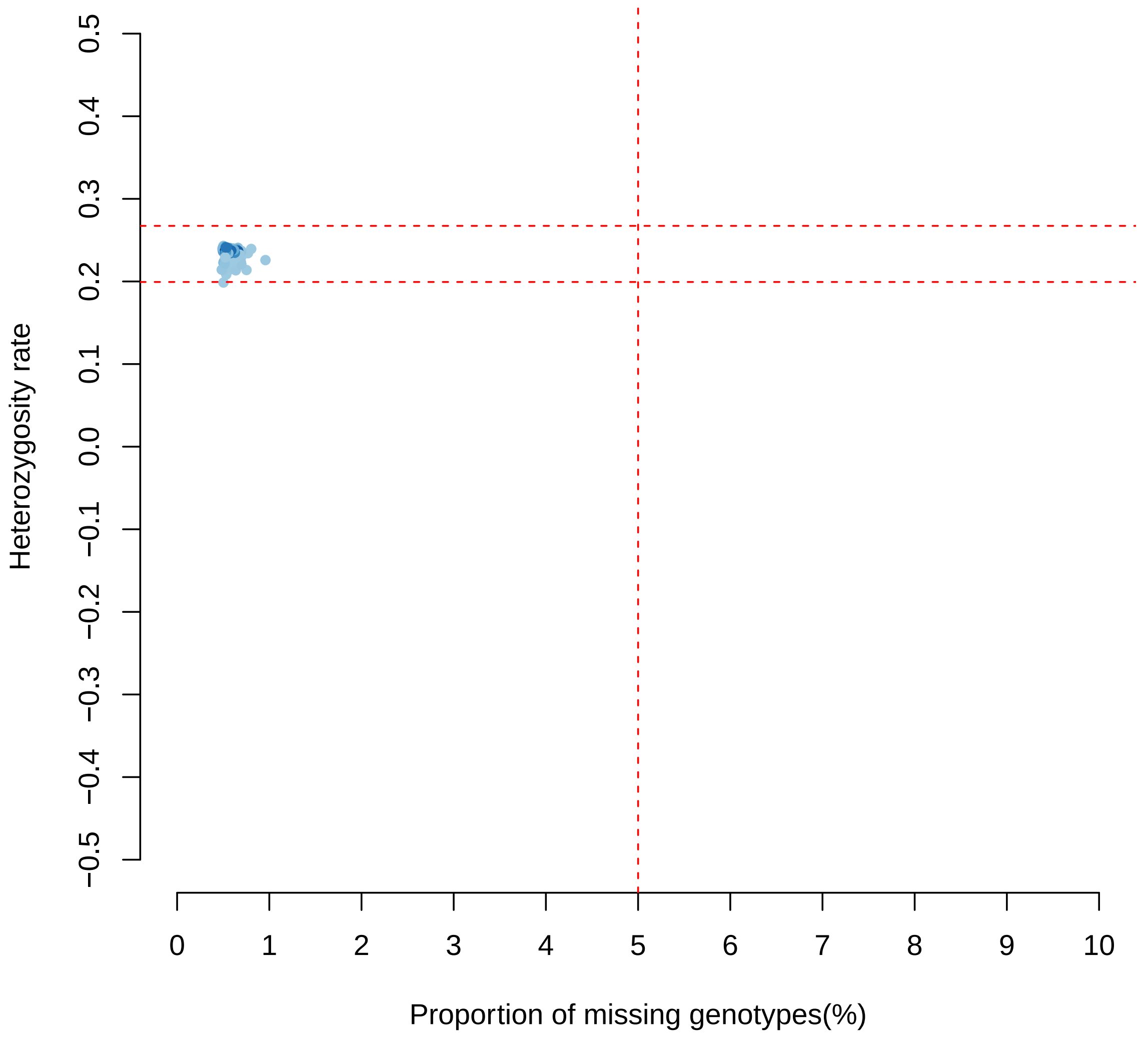

Figure 1: Missing genotype and heterozygosity

1.1.2 Genotyping rate distribution

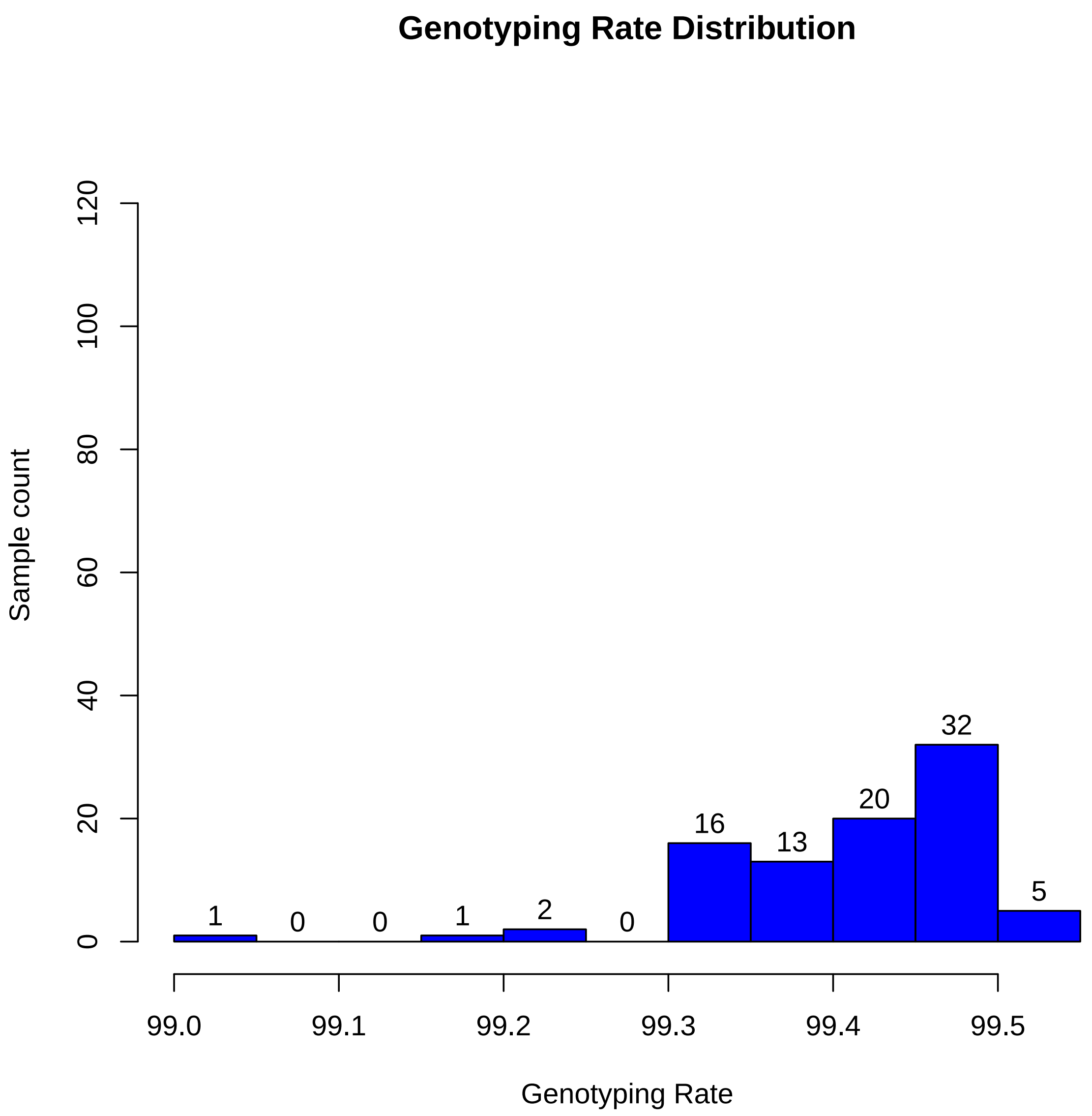

Figure 2: Genotyping rate distribution

1.1.3 Principal Component Analysis

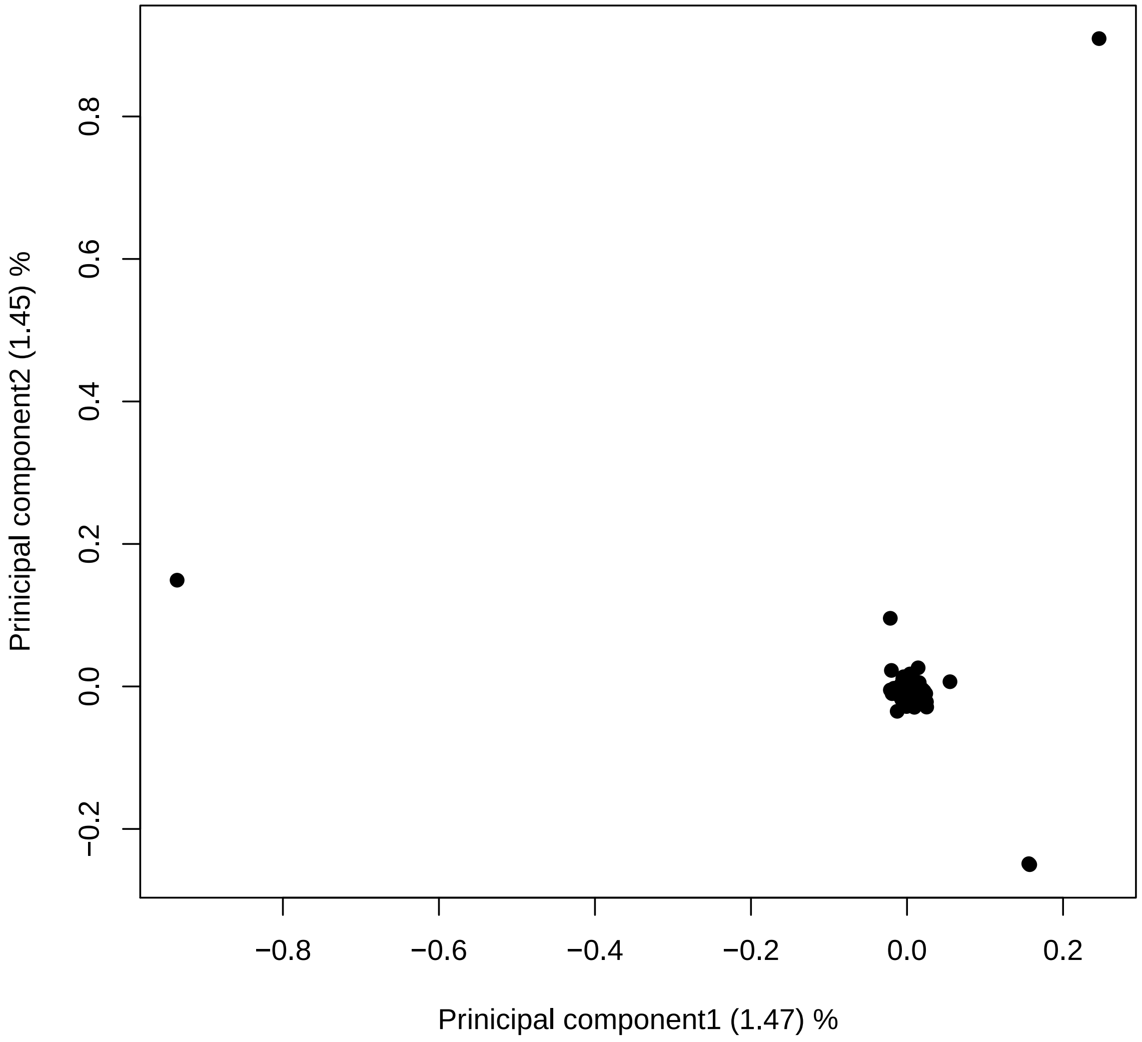

Figure 3: Prinicipal Component Analysis

1.1.4 Genotype Rate Distribution table

Table 1: Genotype Rate Distribution table

Sample_Id	Gender	Phenotype	Genotyping_Rate
7931854	NA	-9	99.43
7931855	NA	-9	99.43
7931856	NA	-9	99.44
7933369	NA	-9	99.45
7933370	NA	-9	99.46
7933371	NA	-9	99.45
7933372	NA	-9	99.45
7933373	NA	-9	99.48
7945593	NA	-9	99.38
7945594	NA	-9	99.39
7956061	NA	-9	99.39
7956062	NA	-9	99.43
7970059	NA	-9	99.44
7970060	NA	-9	99.45
8001562	NA	-9	99.47
8013017	NA	-9	99.43
8013018	NA	-9	99.45
8016531	NA	-9	99.41
8021068	NA	-9	99.45
8025851	NA	-9	99.30
8025854	NA	-9	99.33
8025855	NA	-9	99.36
8025856	NA	-9	99.41
8037295	NA	-9	99.44
8037299	NA	-9	99.43
8037305	NA	-9	99.44
8372850	NA	-9	99.36
8372852	NA	-9	99.33
8372853	NA	-9	99.25
8372854	NA	-9	99.30
8372855	NA	-9	99.31
8372856	NA	-9	99.35
8372857	NA	-9	99.20
8372858	NA	-9	99.38
8372859	NA	-9	99.23
8372860	NA	-9	99.35
8372861	NA	-9	99.34
8372862	NA	-9	99.33
8372907	NA	-9	99.40
8372908	NA	-9	99.35
8372909	NA	-9	99.39
8372910	NA	-9	99.37
8372911	NA	-9	99.34
8372912	NA	-9	99.35
8372913	NA	-9	99.30
8372914	NA	-9	99.33
8372915	NA	-9	99.31
8373005	NA	-9	99.34
8373006	NA	-9	99.32
8373007	NA	-9	99.41
8373008	NA	-9	99.42
8373009	NA	-9	99.43
8373010	NA	-9	99.42
8373039	NA	-9	99.42
8373040	NA	-9	99.04
8373041	NA	-9	99.37
8373042	NA	-9	99.37
8414317	NA	-9	99.50
8414318	NA	-9	99.50
8414319	NA	-9	99.48
8414320	NA	-9	99.49
8414321	NA	-9	99.49
8414322	NA	-9	99.50
8414323	NA	-9	99.49
8414324	NA	-9	99.48

Continued on next page

Table1 *Continued from previous page*

Sample_Id	Gender	Phenotype	Genotyping_Rate
8414325	NA	-9	99.48
8414326	NA	-9	99.48
8414327	NA	-9	99.48
8414328	NA	-9	99.45
8414329	NA	-9	99.51
8414330	NA	-9	99.50
8414331	NA	-9	99.50
8414332	NA	-9	99.49
8414333	NA	-9	99.50
8414334	NA	-9	99.49
8414335	NA	-9	99.48
8414336	NA	-9	99.48
8414337	NA	-9	99.41
8414338	NA	-9	99.38
8414339	NA	-9	99.41
8414340	NA	-9	99.44
8414341	NA	-9	99.52
8414342	NA	-9	99.51
8541290	NA	-9	99.48
8541291	NA	-9	99.48
8541292	NA	-9	99.47
8541293	NA	-9	99.46
8541294	NA	-9	99.48
8541295	NA	-9	99.48
8541296	NA	-9	99.47
